# Primary mitochondrial myopathy: 12-month follow-up results of an Italian cohort

**DOI:** 10.1007/s00415-022-11324-3

**Published:** 2022-08-18

**Authors:** V. Montano, P. Lopriore, F. Gruosso, V. Carelli, G. P. Comi, M. Filosto, C. Lamperti, T. Mongini, O. Musumeci, S. Servidei, P. Tonin, A. Toscano, G. Primiano, M. L. Valentino, S. Bortolani, S. Marchet, G. Ricci, A. Modenese, S. Cotti Piccinelli, B. Risi, M. Meneri, I. G. Arena, G. Siciliano, Michelangelo Mancuso

**Affiliations:** 1 Department of Clinical and Experimental Medicine, Neurological Clinic, University of Pisa, Pisa, Italy; 2 IRCCS Istituto delle Scienze Neurologiche di Bologna, Programma di Neurogenetica, Bologna, Italy; 3 Department of Biomedical and Neuromotor Sciences (DIBINEM), University of Bologna, Bologna, Italy; 4 Department of Pathophysiology and Transplantation (DEPT), Dino Ferrari Centre, University of Milan, Milan, Italy; 5 Fondazione IRCCS Ca’ Granda Ospedale Maggiore Policlinico, Neuromuscular and Rare Disease Unit, Milan, Italy; 6 Department of Clinical and Experimental Sciences, NeMO-Brescia Clinical Center for Neuromuscular Diseases, University of Brescia, Brescia, Italy; 7 UO Medical Genetics and Neurogenetics, Fondazione IRCCS Istituto Neurologico C.Besta, Milan, Italy; 8 Neuromuscular Unit, Department of Neurosciences, University of Torino, Turin, Italy; 9 Department of Clinical and Experimental Medicine, UOC Neurologia e Malattie Neuromuscolari, University of Messina, Messina, Italy; 10 Fondazione Policlinico Universitario A. Gemelli IRCCS, Rome, Italy; 11 Dipartimento Universitario di Neuroscienze, Università Cattolica del Sacro Cuore, Rome, Italy; 12 Department of Neurosciences, Biomedicine and Movement Sciences, Section of Clinical Neurology, University of Verona, Verona, Italy; 13 Neurorehabilitation Unit, Department of Neuroscience, Biomedicine and Movement Sciences, University of Verona, Verona, Italy

**Keywords:** Mitochondrial disorders, Primary mitochondrial myopathy, Outcome measures, 6MWT, Fatigue, Pain

## Abstract

**Objectives:**

To assess natural history and 12-month change of a series of scales and functional outcome measures in a cohort of 117 patients with primary mitochondrial myopathy (PMM).

**Methods:**

Twelve months follow-up data of 117 patients with PMM were collected. We analysed the 6-min walk test (6MWT), timed up-and-go test (× 3) (3TUG), five-times sit-to-stand test (5XSST), timed water swallow test (TWST), and test of masticating and swallowing solids (TOMASS) as functional outcome measures; the Fatigue Severity Scale and West Haven-Yale Multidimensional pain inventory as patient-reported outcome measures. PMM patients were divided into three phenotypic categories: mitochondrial myopathy (MiMy) without extraocular muscles involvement, pure chronic progressive external ophthalmoplegia (PEO) and PEO&MiMy. As 6MWT is recognized to have significant test–retest variability, we calculated MCID (minimal clinically important difference) as one third of baseline 6 min walking distance (6MWD) standard deviation.

**Results:**

At 12-month follow-up, 3TUG, 5XSST and FSS were stable, while TWST and the perceived pain severity (WHYMPI) worsened. 6MWD significantly increased in the entire cohort, especially in the higher percentiles and in PEO patients, while was substantially stable in the lower percentile (< 408 m) and MiMy patients. This increase in 6MWD was considered not significant, as inferior to MCID (33.3 m). NMDAS total score showed a slight but significant decline at 12 months (0.9 point). The perceived pain severity significantly worsened. Patients with PEO performed better in functional measures than patients with PEO&MiMy or MiMy, and had lower values of NMDAS.

**Conclusions:**

PMM patients showed a slow global decline valued by NMDAS at 12 months; 6MWT was a more reliable measurement below 408 m, substantially stable at 12 months. PEO patients had better motor performance and lower NMDAS than PEO&MiMy and MiMy also at 12 months of follow-up.

**Supplementary Information:**

The online version contains supplementary material available at 10.1007/s00415-022-11324-3.

## Introduction

As defined by an international consortium in 2017 [1], “primary mitochondrial myopathies (PMM) are genetically defined disorders leading to defects of oxidative phosphorylation affecting predominantly, but not exclusively, skeletal muscle”. In our previous study, we characterized the functional scales and biomarkers of an Italian cohort of 118 PMM [2]. PMM natural history and evolution over time of outcome measures are unknown; a better knowledge of PMM natural history is of pivotal importance also considering current and future PMM trials. We have therefore collected, basally and at 12 months of follow-up, data of the functional scales already defined in the previous study, trying to: (1) define their evolution over time; (2) trace disease trajectories; (3) explore genotype—phenotype correlations.

## Patients and methods

We have collected the clinical data, the outcome measures and the quality-of-life questionnaires at 12 months (T1) of 117 adult Italian patients with a diagnosis of PMM due to either mtDNA or nDNA mutations affecting mitochondrial oxidative phosphorylation, registered in the Nation-wide Italian Collaborative Network of Mitochondrial Diseases. One patient of the original cohort developed Parkinsonism and, thus, was excluded from the cohort. The baseline characterization of the cohort (T0), as well as the used outcome measures and scales, have been published [2]. Here, we are only recalling the outcome measures performed at 12 months:

1. Clinician-reported outcome measures—clinical scales: the Newcastle Mitochondrial Disease Scale for Adults (NMDAS). We have evaluated global scores and the subitems myopathy and exercise intolerance.
2. Functional tests: 6-min walk test (6MWT), triple timed up-and-go test (3TUG), 5X sit-to-stand test (5XSST), timed water swallow test (TWST), and test of masticating and swallowing Solids (TOMASS).
3. Performance outcome measures: spirometry.
4. Patient-reported outcome measures: Fatigue Severity Scale (FSS) and West Haven-Yale Multidimensional Pain Inventory (WHYMPI).

Starting from the clinical observation, we have now differentiated PMM into three phenotypes: mitochondrial myopathy (MiMy) without extraocular muscles involvement, pure chronic progressive external ophthalmoplegia (PEO) and PEO&MiMy. PEO was defined as a pure ocular myopathy with ptosis and progressive ophthalmoplegia without significant objective muscle weakness and/or exercise intolerance (NMDAS subitem myopathy and exercise tolerance both score of 0/5) or multisystem involvement; PEO patients could refer with subjective muscle pain. On the contrary, PEO&MiMy had ptosis and ophthalmoplegia with other features of muscle involvement like muscle weakness, exercise intolerance (NMDAS subitem myopathy and/or exercise tolerance score ≥ 1/5) and other features of myopathic involvement (dysphagia, restrictive respiratory syndrome). MiMy are those patients without ocular myopathy and with other myopathic signs (proximal/distal or axial muscle weakness, isolated exercise intolerance, myoglobinuria triggered by exercise) and/or other signs of myopathic involvement.

Standard protocol approvals, registrations, and patient written informed consent was obtained from all participants, and the ethics committees of each centre approved the study.

### Statistical analysis

Frequencies, average, median, SD, standard error, and percentiles were calculated for each feature. Values were reported as mean ± SD for variables with normal distribution, as median and interquartile range (IQR) for variables with skewed distribution, and as a percentage for categorical data. To verify the distribution of each parameter, the Kolmogorov–Smirnov test was performed. For continuous variables, the independent Student *t* test and Mann–Whitney *U* test (Wilcoxon's test) were applied to find differences between 2 groups. For comparisons between paired data (T0 and T12), we used Student t-test for data with normal distribution and Wilcoxon test for data with non-normal distribution. Proportions were analysed by Fisher’s exact. Differences among patients with the three different phenotypes were evaluated using analysis of variance one-way and Bonferroni post hoc tests for data with normal distribution and Kruskal–Wallis and Dunn-Bonferroni post hoc tests for data with skewed distribution, after assessing the equality of variances for each variable using parametric and nonparametric Levene tests. In all cases, a *p* value of less than 0.05 was regarded as significant; a lower value was indicated if it was found. Biostatistical analysis was performed with IBM SPSS 20.0.0 program.

## Results

The predominant phenotype was PEO&MiMy (44.9%), followed by MiMy (36.4%) and PEO (17.8%) (Table 1), both at baseline and follow-up.

**Table 1:** PMM phenotype distribution

Phenotype distribution	Number of patients	Percentage
Lost at follow-up	1	0.8
PEO	21	17.8
PEO&MiMy	53	44.9
MiMy	43	36.4
Total	118	100.0

*PEO* progressive external ophthalmoplegia, *MiMy* mitochondrial myopathy

**Fig. 1:**
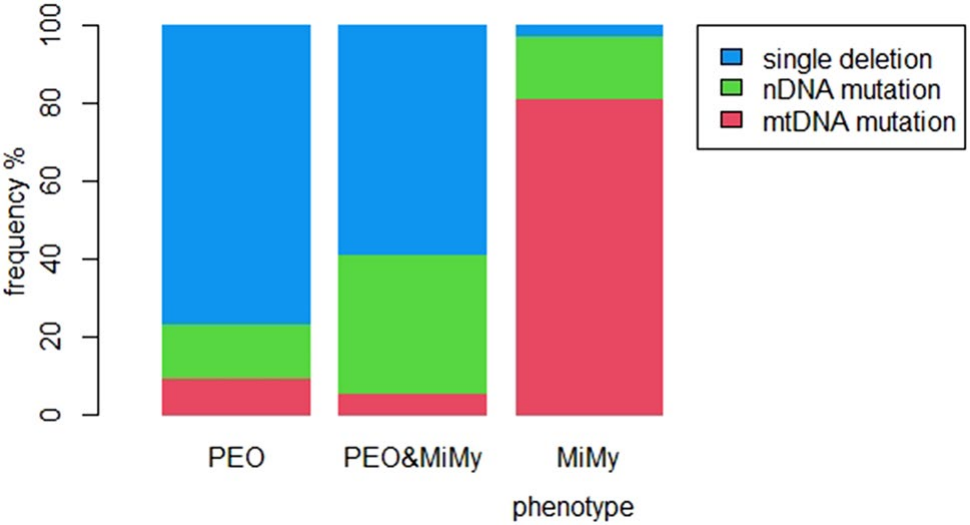
The histogram represents the different proportion of genotypes in the 3 PMM phenotypes. The histogram represents the different proportion of genotypes in the three PMM phenotypes described. *PEO* progressive external ophthalmoplegia, *MiMy* mitochondrial myopathy

PEO and PEO&MiMy presented a significantly higher proportion of single deletion vs MiMy (respectively 76.2% and 58.5% vs 2.3%, both *p* < 0.005), whereas PEO&MiMy exhibited the highest proportion of nuclear DNA mutation although not statistically significant when compared with the other two phenotypes (47.4% with *POLG* mutations). The predominant genotype of MiMy (81.4%) was a mtDNA point mutation [18 of 35 (51.4%) m.3243A>G and 11 of 35 (31.4%) m.8344A>G]. The prevalence of mtDNA mutations in MiMy were significantly higher than in PEO and PEO&MiMy (respectively 81.4% vs 9.5% and 5.7%, both *p* < 0.005 (Fig. 1). The distributions of mutations in our cohort are shown in supplementary Table 1.

**Fig. 2:**
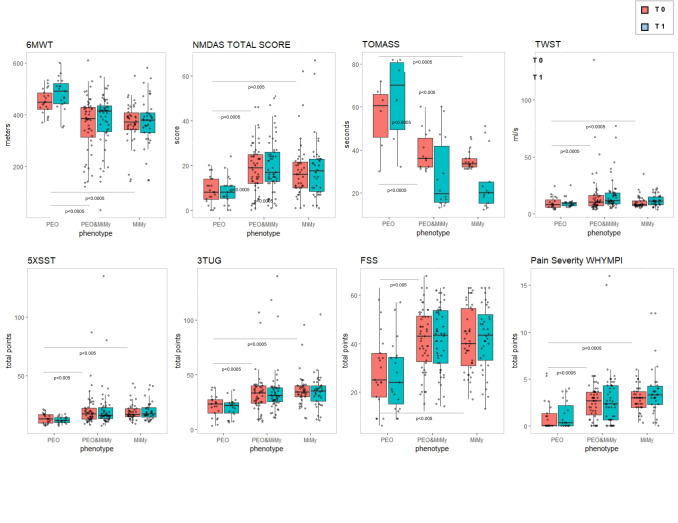
Difference between PMM phenotypes (red plots T0, blue blots T1). *6MWT* 6-min walk test, *3TUG* triple timed up-and-go test, *5XSST* 5X sit-to-stand test, *FSS* Fatigue Severity Scale, *NMDAS* The Newcastle Mitochondrial Disease Scale for Adults, *WHYMPI* West Haven-Yale Multidimensional Pain Inventory, *TWST* timed water swallow test, *TOMASS* test of masticating and swallowing solids

PEO patients showed significantly better performance than PEO&MiMy and MiMy in 6MWT (*p* < 0.0005) and several other outcome measures (3TUG *p* < 0.0005, 5XSST *p* < 0.005, NMDAS total score *p* < 0.005 and subitems *p* < 0.0005) and less fatigue and pain (FSS *p* < 0.005 and WHIMPY *p* < 0.0005), both at T0 and T1 (Fig. 2). PEO&MiMy and MiMy did not differ significantly in functional outcome measures results.

Table 2 shows the paired data (T0 and T1) for each functional outcome measure in the whole cohort. NMDAS total score and its subitems (myopathy and exercise intolerance), TWST, TOMASS and pain severity showed a significant worsening, while apparently in the entire cohort 6MWD (6 min walking distance) seems to show a significant improvement (17.98 m). NMDAS total score, subitem myopathy and exercise tolerance declined from T0 to T1 of 0.9, 0.43 and 0.33 respectively.

**Table 2:** Distribution of paired values in T0 and T1 evaluation for all patients

	*N°* patients	Average	SD	Percentiles			*p* value
				25°	50°(median)	75°	
6MWT T0	116	375.37	104.28	342.25	390.00	437.75	**0.006**
6MWT T1	107	393.35	102.95	349.00	408.00	460.00	
3TUG T0	114	32.26	17.51	22.75	31.69	39.00	0.552
3TUG T1	107	32.49	20.10	22.41	30.32	38.00	
TOMASS T0	39	40.05	11.19	32.00	36.00	46.00	**0.001**
TOMASS T1	33	33.33	21.61	16.00	23.00	47.50	
5XSST T0	107	18.58	10.32	13.00	16.00	20.83	0.763
5XSST T1	106	19.37	14.88	12.40	15.97	21.00	
TWST T0	103	12.97	15.30	7.00	8.68	14.00	**0.001**
TWST T1	96	13.99	11.14	8.00	10.89	16.30	
FSS T0 *	116	39.47	14.73	28.00	39.00	51.75	0.865
FSS T1 *	105	39.37	14.95	28.00	41.00	52.50	
NMDAS Myopathy T0	107	1.08	1.108	0.00	1.00	2.00	**0.00.001**
NMDAS Myopathy T1	110	1.51	1.232	0.00	1.00	2.00	
NMDAS Exercise Tolerance T0	107	1.35	1.047	1.00	1.00	2.00	**0.00.009**
NMDAS Exercise Tolerance T1	110	1.68	1.165	1.00	2.00	2.00	
NMDAS TOTAL SCORE T0	118	16.59	11.175	8.75	15.00	23.00	0.002
NMDAS TOTAL SCORE T1	110	17.67	12.690	8.00	15.00	24.00	
CK (U/L) T0	87	270.79	341.90	118.00	191.00	318.00	0.260
CK (U/L) T1	95	281.97	238.95	120.00	201.00	322.00	
Lactate (mg/dL) T0	98	19.33	11.46	11.13	18.02	23.63	0.052
Lactate (mg/dL) T1	76	20.05	12.03	10.81	15.95	27.64	
FEV1 (%) T0 *	56	84.98	15.61	77.25	84.00	92.75	0.146
FEV1 (%) T1	56	83.03	15.18	74.75	82.50	93.00	
Pain severity WHYMPI T0	112	2.35	1.65	1.00	2.33	3.60	**0.017**
Pain severity WHYMPI T1	100	2.88	2.91	0.66	2.66	4.00	

*T*-student test was used for values with normal distribution (indicated with an asterisk), Wilcoxon's test was used for values with skewed distribution (all the others). In bold the significant *p* values

*6MWT* 6-min walk test, *3TUG* triple timed up-and-go test, *5XSST* 5X sit-to-stand test, *TWST* timed water swallow test, *TOMASS* test of masticating and swallowing solids, *FSS* Fatigue Severity Scale, *NMDAS* The Newcastle Mitochondrial Disease Scale for Adults, *WHYMPI* West Haven-Yale Multidimensional Pain Inventory, *CK* creatine kinase, *FIV* forced inspiratory volume, *SD* standard deviation

In Table 3, we show paired values at T0 and T1 evaluation according to PMM phenotype, and only data with significant variation are shown. 6MWT significantly improved in PEO, but was substantially stable in MiMy and PEO&MiMy.

**Table 3:** Paired values at T0 and T1 evaluation according to phenotype distribution

	*N°* patients	Average	SD	Percentiles			*p* value
				25°	50° (median)	75°	
*PEO population*							
6MWT T0	21	449.6048	48.00855	413.5	447	492.5	
6MWT T1	19	479.8421	65.09759	440	490	530	0.006
TWST T0 *	19	9.5489	5.31091	6	8.07	14	
TWST T1	17	9.9947	4.85.075	6.96	8.85	10.56	0.035
*PEO&MiMy population*							
TOMASS T0	14	39.14	9.189	31.75	36.00	47.50	
TOMASS T1	14	28.29	17.013	15.00	19.50	46.25	0.004
TWST T0	46	16.5472	21.67441	6.9550	10.1650	16.4150	
TWST T1	47	16.9904	14.55371	9.0000	11.4100	19.0000	0.033
NMDAS exercise tolerance T0	49	1.55	0.959	1.00	1.00	2.00	
NMDAS exercise tolerance T1	53	1.89	1.068	1.00	2.00	2.50	0.001
NMDAS TOTAL SCORE T0 *	53	18.91	10.964	12.00	19.00	25.50	
NMDAS TOTAL SCORE T1 *	53	20.09	12.312	12.50	17.00	26.50	0.05
Pain severity WHYMPI T0	51	2.4531	1.61657	1.0000	2.6700	3.6000	
Pain severity WHYMPI T1	47	3.0132	3.22996	0.6600	2.3300	4.3000	0.044
*MiMy population*							
TOMASS T0	18	35.06	4.709	31.75	33.50	36.75	
TOMASS T1	13	24.85	13.631	14.50	20.00	34.50	0.005
TWST T0	37	10.1868	5.61168	7.0000	8.0000	11.6000	
TWST T1	32	11.7141	5.07932	7.9175	11.0000	15.0000	0.052
NMDAS myopathy T0	38	1.24	0.998	0	3	0.00	
NMDAS myopathy T1	38	1.84	1.175	0	5	1.00	0.013
NMDAS exercise tolerance T0	38	1.68	0.989	0	4	1.00	
NMDAS exercise tolerance T1	38	2.11	1.008	0	5	1.00	0.012
Lactate (mg/dL) T0 *	38	19.9058	9.89151	12.5200	20.0600	24.5375	
Lactate (mg/dL) T1 *	25	25.3724	12.92778	13.5150	26.1300	32.4150	0.013

*T*-student test was used for values with normal distribution (indicated with an asterisk), Wilcoxon's test was used for values with skewed distribution (all the others). Only the significant *p* values are shown

To better analyse the apparent increase in the 6MWD, which however, we could explain by a training effect or by the intrinsic variability of the test (discussed later [3]), we conducted a sub-analysis in two ways: by dividing patients by phenotype and by comparing the lower percentiles with the higher ones. Analysing by percentiles (Table 4), we found a stability of 6MWT in the lower percentiles and a significant 6MWT increase in the two higher percentiles. The lower and the higher two percentiles had a different distribution of phenotypes: the higher percentiles showed significantly more PEO patients (most of them with single deletion) and significantly fewer MiMy (Table 5).

**Table 4:** Paired values in T0 and T1 evaluation according to median of results obtained by patients in 6MWT in T1

	*N°* patients	Average	SD	Percentiles			*p* value
				25°	50° (median)	75°	
*Patients with 6MWT ≤ 408M*							
6MWT T0	54	318.2750	94.75411	262.7500	345.0000	389.0000	
6MWT T1	54	318.7648	86.12732	297.5000	349.5000	381.2500	0.350
TOMASS T0	18	37.50	7.778	31.75	36.00	39.50	
TOMASS T1	18	25.67	14.430	17.00	20.50	26.75	0.0005
NMDAS myopathy T0	49	1.63	1.112	1.00	2.00	2.50	
NMDAS myopathy T1	54	2.02	1.073	1.00	2.00	3.00	0.0004
NMDAS exercise tolerance T0	49	1.84	0.986	1.00	2.00	2.50	
NMDAS exercise tolerance T1	54	2.20	1.016	1.75	2.00	3.00	0.0006
NMDAS TOTAL SCORE T0 *	54	18.61	11.635	9.50	20.50	24.25	
NMDAS TOTAL SCORE T1 *	54	20.13	12.842	9.50	21.50	25.25	0.012
Pain severity WHYMPI T0	50	2.95	1.33	2.00	3.00	4.00	
Pain severity WHYMPI T1 *	47	3.43	2.49	2.30	3.300	4.33	0.052
*Patients with 6MWT > 408M*							
6MWT T0 *	53	440.1038	59.06038	403.0000	435.6000	474.2500	
6MWT T1 *	53	469.3396	48.79363	425.0500	460.0000	506.0000	0.000025
TWST T0	50	9.9778	7.75935	6.0000	7.0850	12.2275	
TWST T1	48	11.6165	7.50887	7.0000	9.0000	15.3000	0.005
NMDAS myopathy T0	49	0.57	0.764	0.00	0.00	1.00	
NMDAS myopathy T1	53	0.87	0.981	0.00	1.00	2.00	0.005
NMDAS exercise tolerance T0	49	0.82	0.782	0.00	1.00	1.00	
NMDAS exercise tolerance T1	53	1.02	0.843	0.00	1.00	2.00	0.033

*T*-student test was used for values with normal distribution (indicated with an asterisk), Wilcoxon's test was used for values with skewed distribution (all the others)

**Table 5:** The table shows the frequency of PMM phenotypes in the lower two percentiles (6MWT < 408M) and in the higher two percentiles (6MWT > 408M)

	≤ 408M	> 408M	*p* value
PEO	2 (3.7%)	17 (32.1%)	< 0.0001
PEO&MiMy	24 (44.4%)	27 (50.9%)	Ns
MiMy	28 (51.9%)	9 (17%)	< 0.0001
Total	54	53	

Proportions were analyzed by Fisher’s exact

*Ns* not significant, *PEO* progressive external ophthalmoplegia, *MiMy* mitochondrial myopathy

**Fig. 3:**
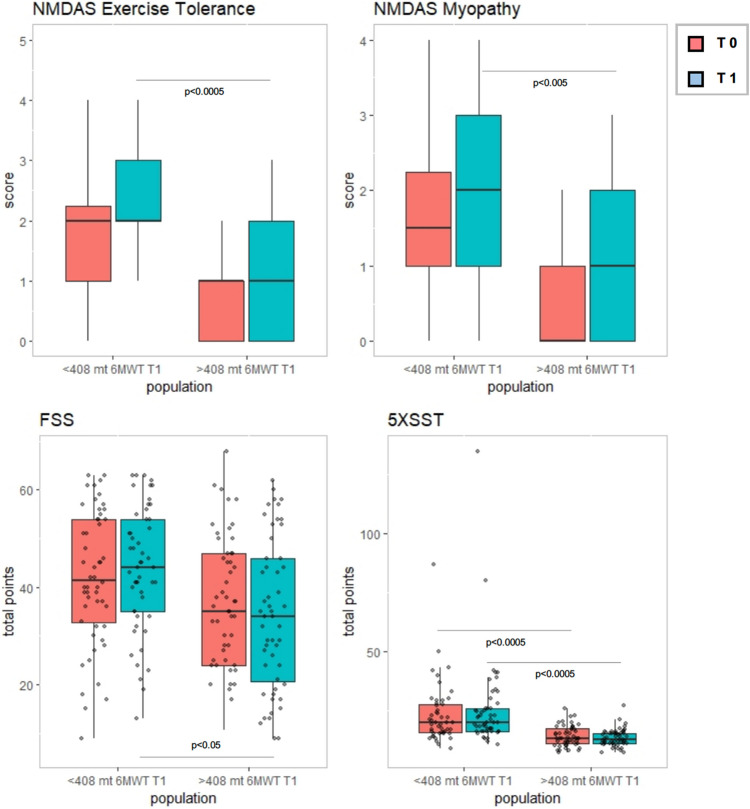
Differences in NMDAS subitem, 5XXST and FSS between higher and lower percentiles based on T1 6MWT (T0 red plot, T1 blue plot). 5X sit-to-stand test, *FSS* Fatigue Severity Scale, *NMDAS* The Newcastle Mitochondrial Disease Scale for Adults, *6MWT* 6-min walk test

Furthermore, the lower percentiles showed worst score on both NMDAS subitem myopathy and exercise tolerance, worst score on 5XSST (Fig. 3).

We therefore hypothesized that the apparent increase of 6MWD in the whole cohort could be explained by the increased variability of 6MWD in the PEO phenotype and in patients with better physical performance.

**Fig. 4:**
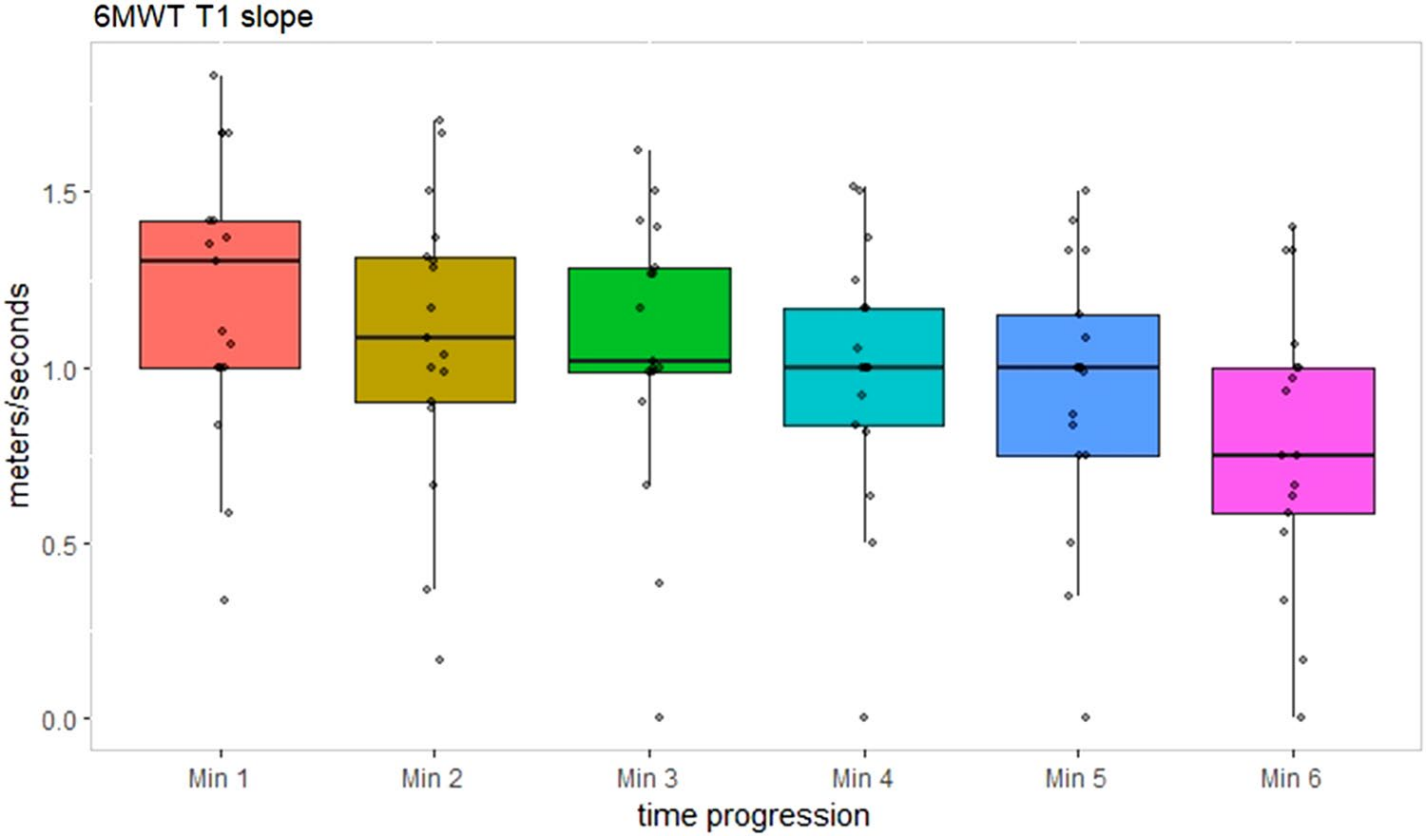
6MWT slope: in 17 patients, we observed a significant decline in 6MWT speed (m/s) between the first and the last minute. *6MWT* 6-min walk test

In a subset of 17 patients (at T1), we have assessed the 6MWT fatigability (slope) (first and last minute speed comparison): the comparison was statistically significant (first minute 72.7 ± 24.3 m vs last minute 47.5 ± 24 m; mean speed 1.2 m/s first minute vs 0.8 m/s last minute, *p* < 0.005) (Fig. 4).

6MWT is recognized to have significant test–retest variability, so, as previously done for other neuromuscular disorders like Pompe disease or Duchenne muscular dystrophy [4, 5], we calculated the MCID (minimal clinically important difference) [6]: baseline SD/3; MCID for the whole cohort was 33.3 m.

**Fig. 5:**
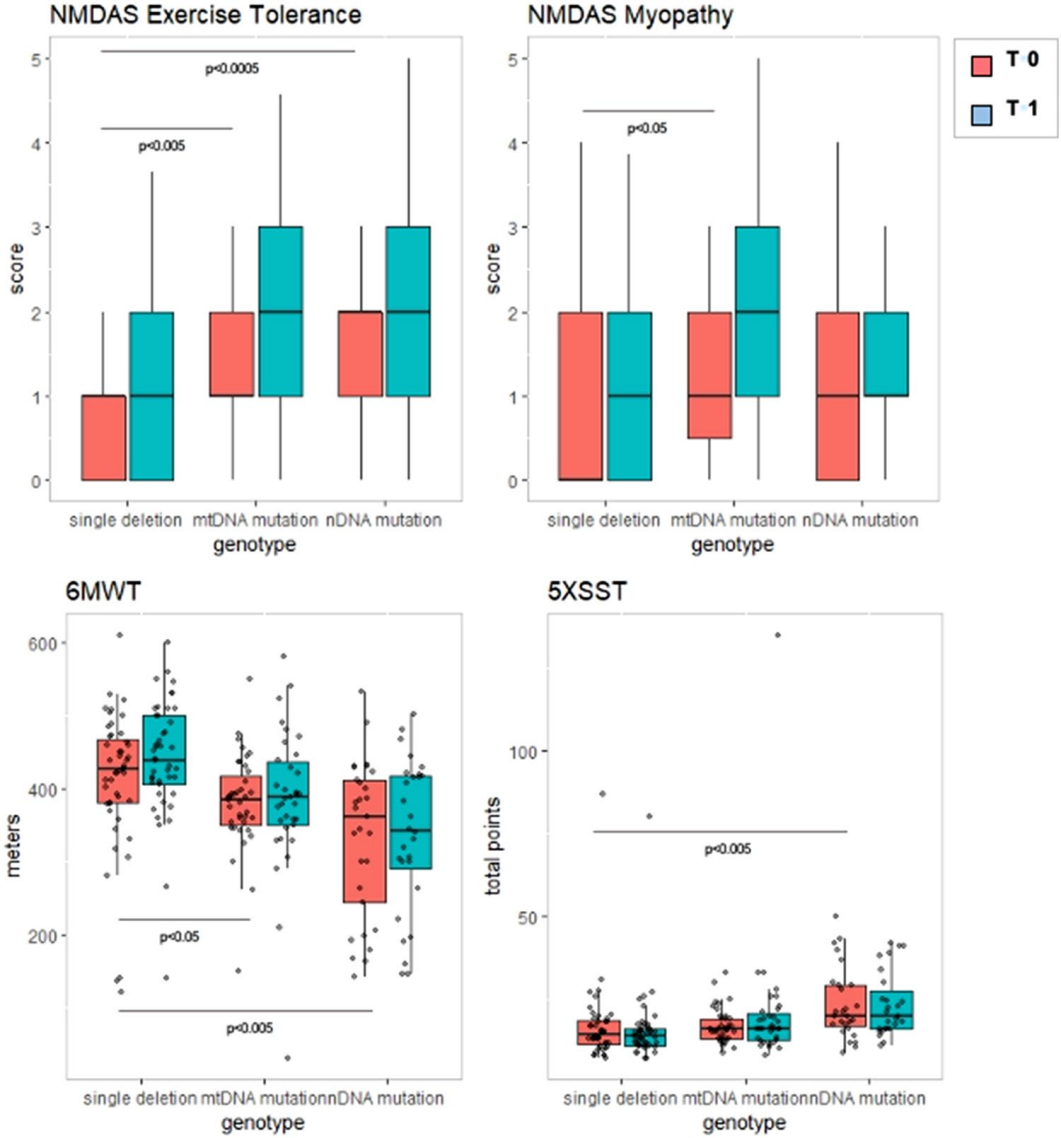
Differences among genotypes. T0 red plot, T1 blue plot. *6MWT* 6-min walk test, *5XSST* 5X sit-to-stand test, *NMDAS* The Newcastle Mitochondrial Disease Scale for Adults, *mtDNA* mitochondrial DNA, *nDNA* nuclear DNA

Differences found at baseline on mtDNA, nuclear DNA and single deletion [2] were confirmed at follow-up; single deletion showed better performance on 6MWT but worst score of 5XSST when compared with nuclear DNA mutations; NMDAS total score was not significantly different between genotypes, mtDNA single deletion had better NMDAS subitem exercise tolerance vs nDNA and other mtDNA mutations and better NMDAS subitem myopathy vs mtDNA mutation (Fig. 5).

## Discussion

We described the 12-month evolution of selected outcome measures in a cohort of 117 PMM patients. The three PMM phenotypes showed a different proportion of genotypes: we confirm the previous observation of a positive association between ocular myopathy (both PEO and PEO&MiMy) with single mtDNA deletion and nuclear DNA (mainly POLG) mutations [7], while MiMy phenotype is associated with other mtDNA mutations, mainly m.3243A>G and m.8344A>G.

The three PMM phenotypes also showed significant differences in outcome measures, as PEO had significantly better value of 6MWT compared to MiMy and PEO&MiMy. Moreover, PEO patients showed significantly better performance than PEO&MiMy and MiMy in other outcome measures (3TUG, 5XSST, NMDAS total score and subitems) and less fatigue and pain, at both T0 and T1. PEO patients walked more at the 6MWT at 12 months follow-up, while in MiMy and PEO&MiMy this was not observed. In the whole cohort NMDAS total score, subitem myopathy and exercise tolerance declined from T0 to T1 of 0.9, 0.43 and 0.33, respectively, whereas these declines are not evident in PEO.

It is difficult to compare our data with those in the literature, as the published series have shorter follow-up times. Madsen et al. in the MOTOR trial stated a “large interparticipant and intraparticipant variation”, even in the placebo group, with a significant variation (up to 130 m in two placebo treated PMM) [8]; moreover, the 6MWD median value was high (442 ± 133 in the placebo group, 421 ± 130 in the omaveloxone group) and similar to our two higher percentiles. A similar 6MWD increment was also described in the elamipretide trial, in which an improvement of 20.9 m was observed in the placebo group at 5 days [9].

The interparticipant and intraparticipant variability of 6MWD is well known; 6MWT has a significant variability across different tests and observers, both in healthy people and in several diseases, including PMM [3, 10]. On the other hand, the 6MWT has been used as the main outcome measure in several trials of neuromuscular diseases, including Pompe disease, mucopolysaccharidosis, Duchenne dystrophy and spinal muscular atrophy [6, 11–14].

Its role in assessing motor performance and exercise intolerance has been evaluated in PMM, and some researchers proposed other measures, like 12MWT or 6MWT slope across minutes as described in RYR related myopathies [10, 15]. Flickinger et al. recently showed firstly 6MWT slop as a measure of exercise intolerance [10]. Our data, but also the MOTOR trial, showed a great variability in 6MWT, in our cohort especially in the higher percentiles with more PEO patients and single deletion. On the other hand, the 6MWT in the lower two percentiles was stable at 12 months.

In the whole cohort, the apparent increase in 6MWD (17.98 m) is striking; however, this increase is lower than the MCID (33.3 m). The MCID is defined as “the smallest difference in score in the domain of interest which patients perceive as beneficial and which would mandate, in the absence of troublesome side effects and excessive cost, a change in patient management” [16]. Therefore, this increase in 6MWT is probably not to be considered clinically significant. Under this consideration, we can affirm a substantial stability of the 6MWT at 12 months.

Based on our data, we consider useful for a clinical trial set-up our clinical and genetic subgroup PMM classification. Although some genotype–phenotype correlations were observed, these are not as strong as is often the case in mitochondrial medicine; therefore, it would be advisable to evaluate the effect of therapies on specific phenotypes rather than specific genotypes.

We did not find any difference between MiMy and PEO&MiMy populations in the analysed outcome measures; however, we do believe that MiMy, with or without PEO, should not be considered as a single entity in future clinical trials because, as shown in Table 5, the presence of PEO in the MiMy patients may lead to a milder phenotype as demonstrated by the 6MWD above 408 m in 50% of cases (Table 5).

Overall, 6MWT is a good outcome measure in PEO&MiMy and MiMy walking less than 408 m, but not in PEO and in those walking above 408 m, providing that the primary endpoint of an interventional trial is not the stability at 12 months, which is intrinsic to the natural history of the disease. It would be interesting to evaluate the effect of therapies on the 6MWD slope even if we offer only a few supporting observations. We have no biomarkers (FGF-21 and GDF-15) follow-up data; their role in future trials is still unclear, although in TK2 myopathy a reduction in GDF-15 levels after treatment has recently been observed [17].

Unfortunately, because of the COVID-19 pandemic, we were not able to re-evaluate most of our patients at 24 months, as originally planned. Further natural history studies, with prolonged monitoring of appropriate outcome measures, are needed. As the literature indicates, some PMM patients may also develop additional system or organs involvements, which was the case for our excluded patient developing parkinsonism, and this is a typical feature of primary mitochondrial diseases, impinging not only on their phenotypic classification but, most importantly, changing their responses to specific outcome measures.

This work represents a real-life picture of a cohort of patients with PMM monitored for twelve months, which provides important and useful information for the planning of clinical trials. For example, from this work we may affirm that a clinical trial on PMM cannot have the stability of 6MWT at 12 months as endpoint, since this is intrinsic in the PMM natural history. Furthermore, inclusion criteria that allow the enrollment of all PMM, despite different phenotypes (PEO and PEO&MiMy or MiMy) that evolve differently in a twelve-month period, may lead to a strong methodological bias and failure of the trial.

## Data availability statement

Current article data are accessible from Michelangelo Mancuso, University of Pisa. In accordance with the data protection legislation in Europe (General Data Protection Regulation), to share the data of the Italian Network, it is necessary to stipulate an agreement between the University of Pisa and the applicant institution. Study data can be requested by contacting Michelangelo Mancuso (michelangelo.mancuso@unipi.it).

## Supplementary Information

Below is the link to the electronic supplementary material.

Supplementary file1 (DOCX 20 KB)

## Declarations

### Standard protocol approvals, registrations, and patient consents

Written informed consent was obtained from all participants, and the ethics committees of each center approved the study.
