# Supplementary material for: Primary mitochondrial myopathy: 12-month follow-up results of an Italian cohort
Source: J Neurol. 2022 Aug 18;269(12):6555–65. doi: 10.1007/s00415-022-11324-3 (PMC9386197; doi:10.1007/s00415-022-11324-3)
Supplement: Supplementary file 1 — Supplementary file1 (DOCX 20 KB) [file 415_2022_11324_MOESM1_ESM.docx]

**Supplementary Table 1:** The table shows the distribution of mutations in our cohort divided by phenotype

|  | ***gene/mutation*** | ***PEO*** | ***PEO&MiMy*** | ***MiMy*** |  |
| --- | --- | --- | --- | --- | --- |
| nDNA mutations | TWNK | 2 | 2 | 1 |  |
|  | POLG |  | 9 | 1 |  |
|  | OPA1 |  | 3 |  |  |
|  | DNA2 |  |  | 1 |  |
|  | DGUOK |  | 1 |  |  |
|  | COQ10 deficiency |  |  | 1 |  |
|  | c.1277G>A |  | 1 |  |  |
|  | ANT1 |  | 1 |  |  |
|  | multiple deletions | 1 | 2 | 3 |  |
| mtDNA mutations | single deletion | 16 | 31 | 1 |  |
|  | m.9399A>G |  |  | 1 |  |
|  | m.8743G>A |  |  | 1 |  |
|  | m.8356T>C |  |  | 1 |  |
|  | m.8344A>G |  | 1 | 11 |  |
|  | m.5865T>C | 1 |  |  |  |
|  | m.5835G>A |  |  | 1 |  |
|  | m.3255G>A |  |  | 1 |  |
|  | m.3251A>G |  | 1 |  |  |
|  | m.3243A>G |  | 1 | 19 |  |
|  | m.15923A>G | 1 |  |  |  |
|  | m.15467A>G |  |  | 1 |  |
